# SABRes: in silico detection of drug resistance conferring mutations in subpopulations of SARS-CoV-2 genomes

**DOI:** 10.1186/s12879-023-08236-6

**Published:** 2023-05-08

**Authors:** Winkie Fong, Rebecca J. Rockett, Jessica E. Agius, Shona Chandra, Jessica Johnson-Mckinnon, Eby Sim, Connie Lam, Alicia Arnott, Mailie Gall, Jenny Draper, Susan Maddocks, Sharon Chen, Jen Kok, Dominic Dwyer, Matthew O’Sullivan, Vitali Sintchenko

**Affiliations:** 1 Centre for Infectious Diseases and Microbiology - Public Health, Westmead Hospital, NSW Westmead, Australia; 2 Sydney Infectious Diseases Institute, The University of Sydney, Camperdown, NSW Australia; 3 Centre for Infectious Diseases and Microbiology Laboratory Services, Institute of Clinical Pathology and Medical Research, NSW Health Pathology, Westmead, NSW Australia

**Keywords:** SARS-CoV-2, COVID-19, Antiviral Resistance, Bioinformatics, Microbial Genomics

## Abstract

The emergence of resistance to antiviral drugs increasingly used to treat SARS-CoV-2 infections has been recognised as a significant threat to COVID-19 control. In addition, some SARS-CoV-2 variants of concern appear to be intrinsically resistant to several classes of these antiviral agents. Therefore, there is a critical need for rapid recognition of clinically relevant polymorphisms in SARS-CoV-2 genomes associated with significant reduction of drug activity in virus neutralisation experiments. Here we present SABRes, a bioinformatic tool, which leverages on expanding public datasets of SARS-CoV-2 genomes and allows detection of drug resistance mutations in consensus genomes as well as in viral subpopulations. We have applied SABRes to detect resistance-conferring mutations in 25,197 genomes generated over the course of the SARS-CoV-2 pandemic in Australia and identified 299 genomes containing resistance conferring mutations to the five antiviral therapeutics that retain effectiveness against currently circulating strains of SARS-CoV-2 – Sotrovimab, Bebtelovimab, Remdesivir, Nirmatrelvir and Molnupiravir. These genomes accounted for a 1.18% prevalence of resistant isolates discovered by SABRes, including 80 genomes with resistance conferring mutations found in viral subpopulations. Timely recognition of these mutations within subpopulations is critical as these mutations can provide an advantage under selective pressure and presents an important step forward in our ability to monitor SARS-CoV-2 drug resistance.

**Supplementary Information:**

The online version contains supplementary material available at 10.1186/s12879-023-08236-6.

## Introduction

Whilst antiviral therapeutics to protect against severe COVID-19 disease have been an important tool in the control of associated morbidity and mortality, there is mounting evidence of antiviral resistance developing against these agents [1–4]. Several mutations have been identified during pre-clinical in vitro testing of novel or repurposed drugs prior to registration as a therapeutic agent [5, 6]. These experiments are accompanied by studies that introduce mutations to drug binding sites to uncover resistance conferring mutations (RCM) [5, 6]. The presence of these RCM or similar mutations can then be investigated in genomes recovered from patients failing treatment with anti-SARS-CoV-2 agents in order to understand why the antivirals are not effective. However, such in silico analyses remain a challenge due to lack of appropriate tools and methodologies. Not surprisingly, our understanding of the prevalence of antiviral RCM in different SARS-CoV-2 variants remains limited. Furthermore, most publicly available SARS-CoV-2 genomic datasets are based on consensus genomes which make investigations of viral subpopulations difficult, particularly identifying SARS-CoV-2 within-host evolution under drug selection pressure [6].

Given the rapid rate of SARS-CoV-2 evolution and adaptation to changing environments, it appears that RCM can both be inherently encoded by the infecting SARS-CoV-2 lineage or acquired de novo due to selective pressure induced by drug use in an individual [1–4]. Noteworthy, de novo mutations are increasingly being detected in immunocompromised individuals and patients at risk for severe COVID-19 disease, the target population for SARS-CoV-2 antiviral treatments [2–4, 7]. Indeed, the variant of concern (VOC) Omicron sub-lineages BA.1 and BA.2 have accumulated a large number of mutations in the Spike gene and appear to be intrinsically resistant to almost all licensed monoclonal antibodies (mAb) targeting the spike protein receptor-binding domain (RBD) [8]. As a result, the remaining drugs at the time of writing, that retain activity against Omicron are Sotrovimab (BA.1 only), Bebtelovimab, Remdesivir, Paxlovid (combination of Nirmatrelvir and Ritonavir) and Molnupiravir [8].

**Fig. 1:**
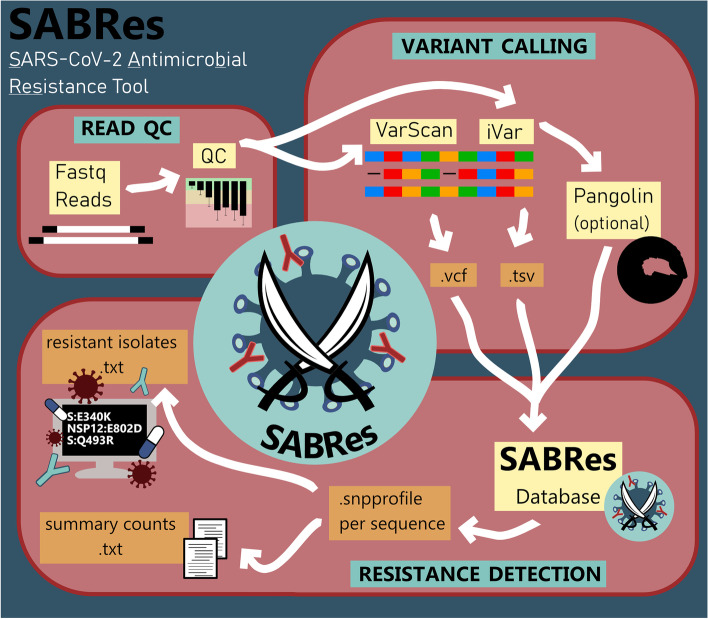
Workflow of the bioinformatics utilised to screen genomes using SABRes for resistance-conferring mutations at the consensus and sub-consensus level

In this study, we present SABRes, a bioinformatic tool that can systematically scan large numbers of files containing SARS-CoV-2 positional mutation data (e.g., variant calling files) for antiviral RCM (Fig. 1). This tool can also detect sub-consensus mutations in viral subpopulations. SABRes enables a rapid snapshot of viral subpopulations that may be selected for when SARS-CoV-2 infected patients are treated with a particular agent. For example, SABRes can detect mutations at low frequencies, rather than at the consensus level (> 90% read frequency) identifying previously undetectable Sotrovimab [1] and Remdesivir [3] de novo resistance, which can develop into consensus level mutations within individual hosts due to selective antiviral pressure.

## Results

**Fig. 2:**
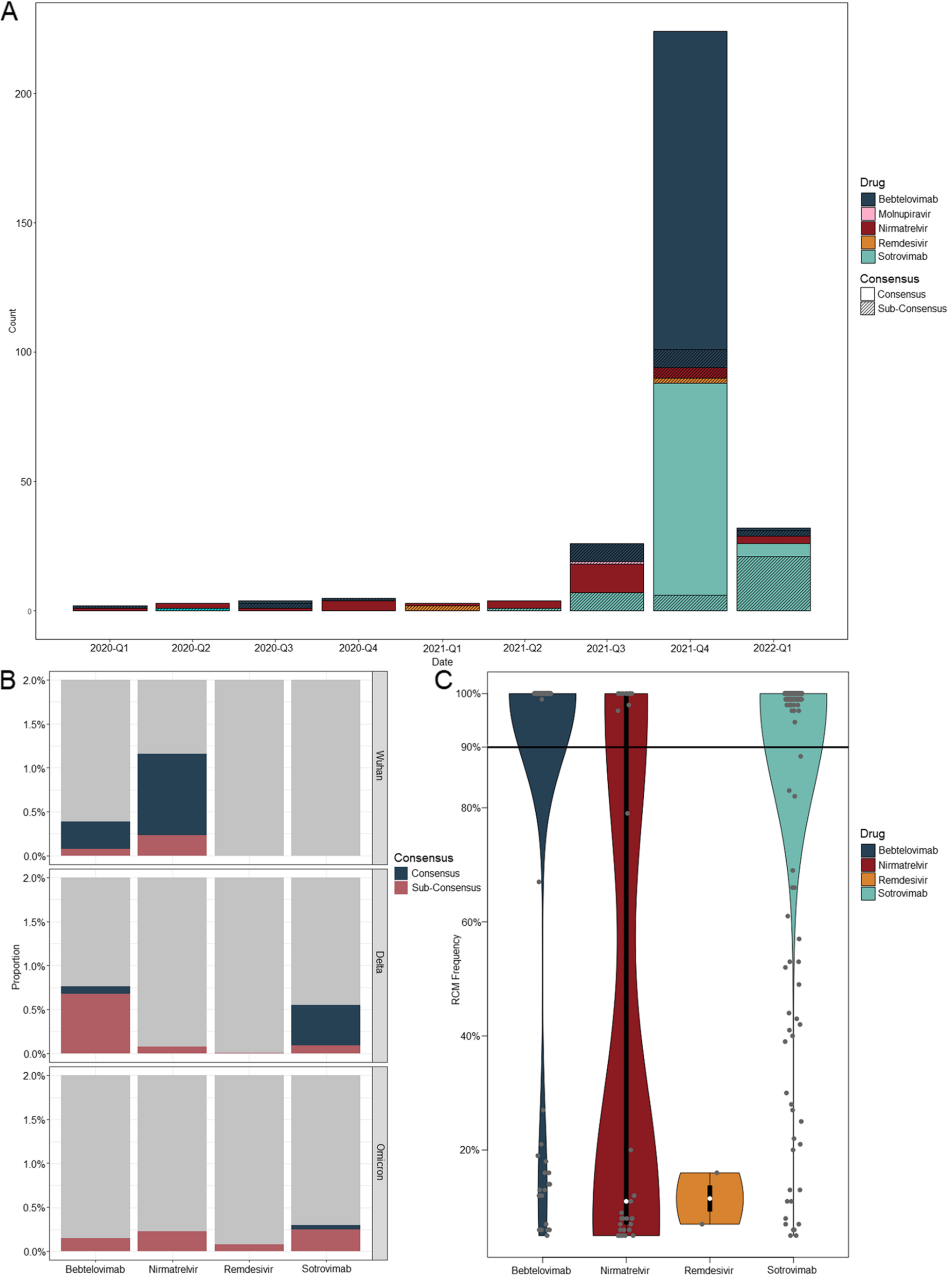
**A** Distribution of resistance conferring mutations (RCM) detected by SABRes between March 2020 and April 2022 (represented in the figure by yearly quarters), excluding the S:S371L/F mutation in BA.1 and BA.2 sub-lineages. **B** The proportion of consensus and sub-consensus RCM from different lineages (original Wuhan strain and two VOC lineages (Delta and Omicron) (maximum y-axis scale of 2% frequency of genomes queried). The S:S371F mutation in BA.1 and BA.2 lineages have been excluded. **C** Violin plot of all detected RCM and distributed based on the sequencing read frequency of the RCM occurring within the sample. Consensus level is denoted at by the black line at 90%. RCM detected against Bebtelovimab and Sotrovimab occurred predominantly at the consensus level (90%), while Remdesivir RCM only occurred at a maximum of 20% read frequency. Molnupiravir data was excluded, as RCM was only detected in a single genome

We applied SABRes to 25,197 SARS-CoV-2 genomes collected between March 2020 and April 2022 from New South Wales (NSW), Australia. Of the 25,197 sequences generated in NSW, Australia, 5,587 (22.17%) genomes carried one or more RCM against the nine licensed SARS-CoV-2 therapeutics screened (Fig. 2A). 3,788/5,587 sequences (67.80%) represented Omicron lineage BA.1 genomes carrying the S:S371L/F mutation which is linked to decreased activity of Sotrovimab where S:S371F is also an Omicron BA.2 lineage defining-mutation.

Twenty-seven genomes contained more than one RCM; 20 of these genomes carried the BA.2/Sotrovimab mutation (S:S371F) (Supplementary Data). The remaining seven isolates contained two or more mutations against Sotrovimab (Fig. 2). When the BA.2/Sotrovimab mutation is excluded, 118/5,584 (2%) genomes contained resistance mutations against Sotrovimab – RCM detected include S:E340A (*n* = 10, 6.69–100% frequency), S:E340K (*n* = 29, 5.01–100% frequency), S:E340V (*n* = 5, 19.72%-100% frequency), S:K356T (*n* = 69, 42.22–100% frequency), S:P337L (*n* = 4, 5.23–10.58% frequency), S:P337H (*n* = 1, 39.01%), S:P337R (*n* = 1, 99.52% frequency), and S:P337T (*n* = 1, 100% frequency). Several isolates that were not BA.1 or BA.2 contained the S:S371F mutation (*n* = 4, 7.04–25.17% frequency). A total of 31 genomes carried sub-consensus level Sotrovimab RCM, while 87 genomes occurred at a frequency above 90%.

Thirty-one genomes contained RCM against Nirmatrelvir (Paxlovid); NSP5:Q189K (*n* = 13, 5.01–20.23% frequency), NSP5:T135I (*n* = 1, 5.14% frequency) and NSP5:H172Y (*n* = 2, 7.73–8.13% frequency) (Fig. 2). NSP5:G15S (*n* = 17, 5.28 – 100% frequency) was detected, however, this mutation is a lineage-defining mutation in the Lambda variant. Sub-consensus level RCM against Nirmatrelvir were most common (*n* = 19) compared with 12 genomes with consensus Nirmatrelvir resistance mutations.

Among the known Bebtelovimab RCM, the following were observed across 145 genomes; S:G446D (*n* = 1, 5.13% frequency), S:G446V (*n* = 139, 5.53–100% frequency), S:K444N (*n* = 2, 20.87–26.78% frequency), S:K444T (*n* = 1, 11.93% frequency), S:P499R (*n* = 2, 99.92–100% frequency) (Fig. 2). SABRes identified 20/145 (13.79%) genomes containing sub-consensus level mutations that would not have been detected at the consensus level.

Two genomes obtained from samples collected in 2021 contained RCM against Remdesivir, NSP12:E802D and NSP12:V557L, both of which were sub-consensus level mutations occurring at a frequency between 7.24–15.50%, respectively (Fig. 2). One genome contained the unverified resistance mutation against Molnupiravir (NSP12:V557I), at a low frequency of 7%.

Overall, 80 of the total 5,614 RCM (1.43%) detected were at a sub-consensus frequency (Fig. 2C). Pango lineages were determined for all screened isolates, which included VOCs (Alpha, Beta, Delta, and Omicron), as well as some variants of interest (VOI) (Zeta). RCM were only detected in the original Wuhan lineage, Delta, and Omicron lineages. Lineage distribution of these isolates containing RCM, revealed 20 genomes from the original Wuhan lineage carrying RCM against Bebtelovimab and Nirmatrelvir, however, no RCMs were detected against Molnupiravir, Remdesivir or Sotrovimab (Fig. 2B). For Delta lineages, 257 total genomes contained RCM against the five major antivirals. Within the Omicron lineage, only 23 genomes carried resistance markers outside of the S:S371F (Omicron BA.2) resistance marker.

The presence of mutations conferring resistance was also noted for all agents apart from Remdesivir, prior to their registration in Australia. Sotrovimab was registered for use on August 20, 2021, while only 3 genomes with RCM against Sotrovimab were detected prior to registration, a rapid increase in resistance conferring mutations was noted in Q3 2021 and Q1 2022 (*n* = 115 excluding BA.2 lineage marker S:S371F). Mutations conferring resistance to Nirmatrelvir (*n* = 32) were also present prior to registration on January 20, 2022. Bebtelovimab is yet to be registered for use in Australia however resistance mutations have been detected in the three initial SARS-CoV-2 waves on infection (Delta, *n* = 138, Omicron, *n* = 2, Other lineages, *n* = 5).

## Discussion

Our tool identified RCM across the five remaining drugs that retain susceptibility against SARS-CoV-2 in 299 genomes (excluding S:S371L/F in Omicron variants). This accounted for 1.18% of the total number of genomes screened. The first oral SARS-CoV-2 antiviral, Nirmatrelvir (Paxlovid), has been reported to have reduced activity against SARS-CoV-2 genomes carrying the NSP5 mutation G15S, which was detected in 17 sequences within our dataset [5]. However, this mutation is a lineage-defining mutation in the Lambda (C.37) variant. Based on early research, this mutation did not result in reduced susceptibility to Nirmatrelvir in cell culture experiments [5]. As many of these RCM were derived from in vitro experiments using genetically modified mouse hepatitis virus, it is difficult to confirm the relevant resistance in vivo. The identification of SARS-CoV-2 genomes containing potential resistance mutations by SABRes, can be further examined and validated in in vitro sensitivity experiments with live SARS-CoV-2 or pseudovirus. Importantly, the tool can ascertain the RCM prevalence in the community prior and post-antiviral registration. Here we demonstrated presence of RCM prior to drug registration in Australia, particularly in viral populations at sub-consensus level. These rate of resistance conferring mutations increased, particularly for Sotrovimab post registration as these mutations became advantageous to virus replication under selective pressure. Concerningly resistance conferring mutations to Bebtelovimab have been detected during the circulation of the original (Wuhan) lineage, Delta and Omicron waves in Australia, despite the drug not being registered for use. This highlights the need for surveillance of these mutations, which may increase in prevalence post drug registration and use.

Unfortunately, data on mutations conferring resistance to Molnupiravir was not readily available at the time of publication. However, previous studies of Molnupiravir against other coronaviruses revealed that an NSP12 mutation—V558I in Middle Eastern Respiratory Syndrome coronavirus (MERS-CoV), resulted in a twofold reduction in Molnupiravir susceptibility [15]. This mutation in SARS-CoV-2 is in codon 557 (NSP12:V557) and a G15109A single nucleotide polymorphism would result in the non-synonymous change to isoleucine (I). While no data had been released on this mutation in the context of SARS-CoV-2 at the time of writing, we elected to include this mutation in SABRes.

Some limitations of our approach must be acknowledged. First, SABRes performance will depend on up-to-date catalogues of significant RCM to different classes of currently prescribed antivirals. As new RCM emerge as therapeutics to treat COVID-19 are registered and employed, ongoing internationally harmonised curation and assessment of clinically relevant RCM is warranted. Secondly, this tool has been tested on data from one geographic region. However, our testing dataset contains quality sequencing data representing all major variants of concern of SARS-CoV-2 as our laboratory has consistently sequenced representative proportions of SARS-CoV-2 positive samples over the course of COVID-19 pandemic. Lastly, our method was implemented and tested on one bioinformatic pipeline for SARS-CoV-2 population analyses. Nevertheless, SABRes are scalable to other bioinformatic environments. Thus, our future efforts will include regular updates to incorporate newly discovered antiviral RCM, as well as to introduce capabilities for other variant callers.

In conclusion, SABRes provided an accessible and reliable method that can be used with standard variant calling outputs to uncover RCM in SARS-CoV-2 genomes and subpopulations. Continuous monitoring of SARS-CoV-2 resistance remains critical for the prevention and control of widespread circulation of resistant strains. Given more than 50% of developed antivirals are no longer effective against circulating strains, tools that enable antiviral stewardship are urgently required.

## Methods/implementation

Between March 2020 and April 2022, a total of 25,197 SARS-CoV-2 positive respiratory samples were sequenced by the Microbial Genomics Reference Laboratory, Westmead Hospital, NSW, Australia. These samples were sequenced using several SARS-CoV-2 amplification methods including, ARTIC v3, Midnight and Illumina Respiratory Viral Oglio Panel (RVOP) protocols (Illumina, USA). These sequences were quality filtered with a previously described in-house bioinformatics pipeline. Briefly, reads were quality trimmed using Trimmomatic (v0.36) (minimum read quality score of 20, leading/trailing quality of 5). SARS-CoV-2 genomes were analysed with variant callers iVar [9] and Varscan [10, 11]. Pangolin (database dated 2/2/22) [12] was used to determine lineage. SABRes (https://github.com/LilWinx/Sabres) was then used to scan these genomes for a comprehensive and adaptable list of resistance conferring mutations at a minimum of 5% read frequency. The tool can also optionally include lineage data from Pangolin to highlight resistant mutations inherently encoded and the ability to differentiate acquired resistance. For our results presented in this study, we have manually quality filtered the results for mutations with a minimum depth of 100X.

### Curation of resistance conferring mutations

A list of RCM that have been proposed to result in a > five-fold decrease in susceptibility (as determined by https://covdb.stanford.edu/page/susceptibility-data) were manually curated from research literature and therapeutic product information that describes mutations that confer resistance to current antiviral medications (Table 1 and Supplementary Data). A dynamic list of mutations has been supplied in the Supplementary Data and within the GitHub repository.

**Table 1:** Drugs approved by FDA for treatment of the COVID-19 and three examples of resistance conferring mutations targeted by SABRes

Drug class	Drug name	Brand name	Mechanism of action	Corresponding drug resistance conferring mutations	References
Monoclonal antibodies	Sotrovimab	Xevudy	Prevention of cell entry	S:E340D S:E340K S:P337H	[1, 4]
	Tixagevimab and Cilgavimab	Evusheld	Prevention of cell entry	S:E484A S:Q498R	[8]
	Bebtelovimab	-	Prevention of cell entry	S:K444T S:G446D S:P499R	[6]
	Regdanvimab	Regikrona	Prevention of cell entry	S:N501Y	[8]
	Bamlanivimab and Etesevimab	-	Prevention of cell entry	S:Q493R	[8]
	Casirivimab and Imdevimab	Regen-Cov/ Ronapreve	Prevention of cell entry and viral escape	S:G446S S:E484A	[13]
Synthetic antivirals	Remdesivir	Veklury	Inhibition of RNA polymerase	NSP12:A97V NSP12:V557L NSP12:E802D	[3, 14]
	Nirmatrelvir and Ritonavir	Paxlovid	Inhibition of viral protease	NSP5:T135I NSP5:H172Y NSP5:Q189K	[5]
	Molnupiravir	-	Inhibition of RNA polymerase	NSP12:V557I	[15]

### Screening of genomes

The tool uses variant calling files (VCF) from Varscan and tab separated values (TSV) files from iVar, and screens genomes from the list of nucleotide mutations. Two databases have been provided and can be called using the “–full" flag. This is to exclude drugs that have limited activity against the dominant SARS-CoV-2 VOC Omicron (default).

## Supplementary Information

**Additional file 1.**

## Data Availability

A supplementary data file has been provided with a list of genomes on GISAID.

## Abbreviations

RBD: Receptor binding Domain
RCM: Resistance Conferring Mutations
RVOP: Respiratory Viral Oligo Panel
TSV: Tab Separated Values
VCF: Variant Calling Files
VOC: Variant of Concern
VOI: Variant of Interest
